# Interactions between Struvite and Humic Acid and Consequences on Fertilizer Efficiency in a Nonacidic Soil

**DOI:** 10.1021/acs.jafc.4c05472

**Published:** 2024-09-18

**Authors:** Javier Erro, Iñigo Seminario, José M. García-Mina

**Affiliations:** † Environmental Biology Department. Faculty of Sciences. BIOMA Institute. University of Navarra, c/Irunlarrea, 1, Pamplona 31008, Spain; ‡ Magnesitas Navarra, S.A, Av. Roncesvalles, Zubiri, Navarra 31630, Spain

**Keywords:** recycled fertilizers, sequential extraction, struvite, fertilizer efficiency, humic acid

## Abstract

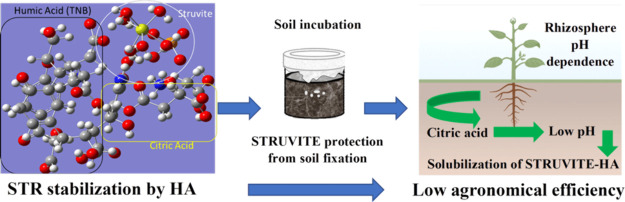

The effect of humic acid extracted from peat (AHt) on improving the struvite (STR) fertilizing efficiency is explored. To this end, a soil incubation study is correlated to plant assays comparing STR, STR-AHt, and superphosphate (SSP). Characterization techniques confirm the incorporation of the AHt into the STR. The P-pool distribution of STR and SSP is similar in the soil incubation, with STR-AHt presenting a higher labile P at 90 days passing from 10 to 15% P from SSP and STR to 25% P with STR-AHt. However, when applied to barley and tomato, STR yields more shoot P content, aboveground biomass, and residual P in soil than SSP. STR-AHt does not improve the STR results. The poor correlation observed between soil incubation and plant trials highlights the role of the rhizosphere in testing the fertilizer efficiency of STR. Mechanistic assays indicate the key role of rhizosphere pH. Finally, molecular modeling reveals a higher stabilization of STR with AHt, which could reduce P release decreasing the fertilizing potential of STR-AHt, as observed in the pot trials.

## 1 Introduction

Phosphorus is one of the three essential nutrients for crop development.^1^ However, its soil bioavailability is relatively low due to interactions with soil.^2,3^ Thus, P inputs in soil are required via P fertilizers. Conventional fertilizers are based on water-soluble P that involves P losses with consequent soil and water pollution. This inefficiency of P fertilization is of great concern as the primary raw material, rock phosphate, is at risk of depletion.^4^ In this context, slow-release fertilizers have been proposed to minimize these handicaps.^5,6^ Nevertheless, slow-release fertilizers nutrient release patterns depend on soil conditions, which involve heterogeneous results.^5^ Another solution is the use of water insoluble but citric-soluble P fertilizers that can provide P to plant roots resulting from its interaction with root exudates in the rhizosphere.^7^ In this line, in the context of circular-economy strategies, several proposals have been described to promote P recycling.^8^ One of them, the use of struvite (STR), a mineral obtained by precipitation of phosphate with magnesium and ammonium in wastewater, can deal with the limitations described above at the same time. It consists of a citric soluble mineral, avoiding P losses in the soil at the time that is adapted to plant needs and is not based on nonrenewable rock phosphate. Thus, it constitutes a green and promising alternative to conventional fertilizers, with the added value of recycling P from polluted wastewater. Moreover, Mg and N in the mineral increase the fertilizing value. Several studies have been carried out testing the agronomic efficiency of STR^9−12^ with heterogeneous results depending on soil pH, crop species, the reference fertilizer, the P application rate, the duration of the assay, and the soil test P on which the experiment is based.^13^ The low solubility of struvite may lead to an insufficient P supply to plants, especially in nonacidic soils with crops of low exudation capacity. Thus, new strategies are required to increase the ability of struvite to release bioavailable P under these conditions. In that way, the role of humic substances (HS) in fertilization practices,^14^ and, especially, in STR properties has recently gained attention. The work of Piri et al.^15^ evaluated the impact on plant growth of STR coprecipitated with leonardite, showing a beneficial effect of this kind of STR related to pure STR. However, to date, the effect of associating HS with crystallized STR on P behavior in soil is scarcely investigated. The majority of the reports are focused on the effect of HS on STR crystallization^15−17^ as its presence decreases STR precipitation by magnesium complexation.

The ability of HS to form stable complexes of P has been widely described.^17,18^ These types of organomineral sources of P present the advantage of protecting P from soil fixation while keeping it available for plant uptake. Therefore, the combination of HS with STR could be a feasible strategy to increase the fertilizer efficiency of STR. In this study, STR is tested in the presence of a humic acid from peat to test the beneficial properties of this modified STR (STR-AHt). The main hypothesis of this work is the increase in the agronomical value of STR by its combination with humic acid in order to obtain organomineral sources of P with a release pattern governed by plant demands. In order to deepen the effect of HS associated with STR in soil P pools, a soil incubation study is developed and compared with pot plant–soil studies. In this way, the effect of STR-AHt is assessed in a dose–response pot assay in nonacidic soil with two crops, barley and tomato, with low exudation capacity in comparison with leguminous, using regrowth harvests in the case of barley and soil analysis after cropping to mimic the long-term effect of STR. Finally, with the aim of delving into the mechanism of action of HS in STR solubilization, an in vitro study of nutrient release from STR and STR-AHt in citrate at different pHs and a computational modeling of STR in the presence of HS and citrate are carried out. Therefore, the study’s objective is to shed new light on the fertilizer efficiency of STR associated with HS in plant assays in a nonacidic soils and correlate these results with soil incubation experiments, *in vitro* solubility tests, and theoretical studies. To this end, the report includes (i) the composition and structural characterization of the STR-AHt, (ii) soil incubation studies of STR-AHt in comparison with STR, diammonium phosphate (DAP), single superphosphate (SSP) and rock phosphate (PR), (iii) a pot trial assay in greenhouse with STR-AHt in comparison with STR and SSP applied at three P doses in tomato and barley, (iv) comparison of P remaining in soil after cropping and regrowth of barley with a sequential P soil test in the incubation study to test the residual effect of the fertilizers, (v) P release kinetics from STR in organic acids at different pHs and P-STR solubility in water in the presence of humic and citrate, and (vi) molecular modeling of STR interactions with humic acid and citrate.

## 2 Materials and Methods

### 2.1 Structural Characterization of the STR and STR-AHt

The wastewater treatment plant E.D.A.R. Cidacos (La Rioja, Spain) provided the STR used in this study. STR was mixed with 20% humic acid from peat (AHt). The humic acid from peat contains 40.1%C and is obtained following the International Humic Substances Society methodology.^19^ STR and AHt were immersed in water for 7 h under stirring to obtain the STR-AHt sample. After centrifugation, the solids were dried and characterized with the following techniques.

#### 2.1.1 FTIR

Functional group distributions in STR and STR-AHt were characterized using ATR-FTIR spectroscopy. Attenuated total reflectance (ATR) infrared spectra were recorded over the 4000–600 cm^–1^ range with a resolution of 4 cm^–1^ in a Shimadzu IRAffinity-1-S. The obtained spectrum was compared with those of soluble (monocalcium phosphate (MCP) and SSP) and insoluble (bicalcium phosphate (BCP) and PR) sources of P used as conventional fertilizers.

#### 2.1.2 X-ray Diffraction

STR and STR-AHt samples’ structure and crystal properties were carried out using X-ray diffractometry. The samples were scanned using a Bruker D8 X-ray powder diffractometer at a starting point of 2.0 2-Theta (deg), end point of 80.0 2-Theta (deg), step-size of 0.020 2-Theta(deg), dwell time of 1 s, and using Cu radiation at 40 kV and 30 mA.

#### 2.1.3 SEM-EDX

STR and STR-AHt were also scanned in a Zeiss model DSM-940A scanning electron microscope (accelerating voltage of 15 kV) with a DISS Point Electronic GmBh image acquisition and processing system. The samples were placed on an adhesive connected with silver and glued to an aluminum support with an earth wire. Before being scanned, the samples were shaded with gold for two min at 15 mA in a vacuum atmosphere using an Emitek K550 Sputter-Coater. Finally, they were photographed at 3000×. For the EDX study, the samples were scanned in a Philips model XL30CP scanning electron microscope (accelerating voltage of 15 kV) using an EDAX Phoenix energy-disperse spectroscopy analyzer. Qualitative elemental composition analysis was carried out on selected areas, which were also photographed at 3000×.

#### 2.1.4 ^31^P-NMR (Nuclear Magnetic Resonance Spectroscopy)

^31^P-NMR spectra were obtained at room temperature by using a Bruker DRX-500 Spectrometer with a static field of 11.7 T. A 1H-BBI probe with a Z gradient was used. Chemical shifts were calibrated with liquid H3PO4 as an external standard. The experiments were performed without sample rotation using a ^31^P pulse at 90° under 1H decoupling on a Waltz16 scheme with a field of 3.13 kHz conditions. The relaxation delay was 3 s, and the number of scans was 256. FID was acquired with 32,000 complex points. All ^31^P spectra were treated with MestreNova software.

### 2.2 Composition of STR and STR-AHt

The concentration of P and Mg of the STR and STR-AHt used in the essays was determined in HCl 35%, in water, and in neutral ammonium citrate 0.96 M according to the EU regulation No. 2003/2003.^20^ P and Mg were determined by ICP-OES spectrometry (Thermo Elemental Co. Iris Intrepid II XDL). The fraction soluble in HCl represents the total content of P and Mg in the fertilizer, whereas the **neutral ammonium citrate** fraction is related to potentially soluble nutrients in the rhizospheric acids exuded by plants in the case of nutrient deficiency. Total N concentration was measured using elemental analysis.

### 2.3 Agronomic Efficiency of STR and STR-AHt

#### 2.3.1 STR and STR-AHt Soil Incubations

A neutral soil was obtained from Eugui (Navarra, Spain). It was sampled from subsurface layers (2 m depth), air-dried, and ground to pass through a 2 mm sieve before analysis. Soil analysis (Table 1) included total C and N via elemental analyzer; P extractable by sodium bicarbonate^21^; K, Mg, Ca, and Na extraction with 1 M ammonium acetate^22^; and Fe, Cu, Zn, and Mn following extraction with dietilentriaminpentaacetic acid (DTPA, pH 7),^23^ to be finally analyzed by ICP-OES. The pH was measured in water (1:2.5 soil/water ratio). For the soil incubation study, 150 g of soil was placed in 300 mL glass pots and treatments (SSP, DAP, PR STR, and STR-AHt) were added and mixed. The P addition rate used was 250 mg of P per kg of soil. A control treatment without phosphorus was added. Finally, soils with treatments were homogenized, and type I deionized water was added to reach the field capacity of the soil previously determined in a soil column, moistened, and allowed to drain freely. Pots were closed and maintained at ambient temperature in the dark for 90 days. Samples were taken and dried in triplicate at 2 and 90 days from the beginning. The P sequential fractionation method proposed by Harrell and Wang^24^ was employed. It comprises the following extractants: 0.5 M sodium bicarbonate (BIC), 0.1 M NaOH, 0.3 M sodium citrate, and 3.3 mL of 1 M sodium bicarbonate (CB), 0.3 M sodium citrate, and 3.3 mL of 1 M sodium bicarbonate with sodium dithionite (CBD) and 1 M HCl. The different fractions were analyzed by ICP-OES. Different fractionation methods have been used with this aim^25^ using resins, BIC, NaOH, and HCl, but the selected method is considered more complete to extract different P-pools in soil.

**Table 1:** Analysis of the Soil Used in the Incubation and Plant Trials

**μg/g soil**								
**Ca**	**K**	**Mg**	**Na**	**P**	**Cu**	**Zn**	**Mn**	**Fe**
662	234	305.1	24.72	5.69	5.72	0.993	12.48	88.31

			**%**					
		**pH**	**organic matter**	**total N**	**lime**	**sand**	**clay**	
		7.26	8.01	0.22	13.77	73.83	12.4	

#### 2.3.2 In Vivo Evaluation of STR and STR-AHt: Pot Essays in Crops

The study was carried out in pots in a glasshouse, with control of light and temperature conditions. The temperature was 24/18 °C day/night, and a relative humidity of 40–60%. A 500 g portion of soil from Eugui was placed in plastic pots, and treatments (SSP, STR and STR-AHt) were added, mixing in a Terminix for 5 s at the maximum power. The P addition rates were 20, 50, and 100 mg of P per kilogram of soil. In that way, a control was added without the addition of external phosphorus. Five pots per treatment and dose were used. Then, it was carefully mixed with 50 g of perlite, and four seeds of tomato or barley, previously germinated in a germination chamber, were transferred to the first centimeter of the soil. Finally, one tomato plant and three barley plants were left per pot. To complete fertilization, 200 mg kg^–1^ of N soil and 200 mg kg^–1^ of soil of K solution (urea + potassium chloride) were added to each pot. Depending on the treatment, the relative amount of Mg introduced with STR and STR-AHt was equilibrated in the other treatments. The pots were maintained weekly at a field capacity. Every 15 days, the NK solution was applied in order to reconstitute nutrients. After 40 days, pots were harvested, and yield and P concentration measurements in dried shoots and the remaining Olsen-P soil fraction were carried out as described below. In the case of barley, two more harvests, at 70 and 100 days from the beginning, were developed after the first one to determine regrowth yields per pot. The dry mass of shoots was determined after drying fresh material in an oven at 40 °C for 3 days, homogenized in a mill, and subsamples were attacked with HNO_3_ and H_2_O_2_ and digested in a microwave to determine P content. The digested sample was analyzed by ICP-OES spectrometry. The P extraction in shoots was calculated by multiplying the P concentration in shoots by aboveground dry weight.

### 2.4 Mechanisms of Action of STR and STR-AHt

#### 2.4.1 Nutrient Release from STR in Different Media

The solubility of STR in sodium citrate (0.96 M) at pH 5.0, 7.0, and 8.5 at different extraction times was studied. To this end, 0.4 g of STR was weighed into 50 mL plastic bottles, and 40 mL of the corresponding extractant was added. The mixture was shaken in a Heidolph Reax 2 model turner at the lowest speed, and aliquots of the solution were taken with a 1 mL syringe at certain times: 5, 10, 15, 30, 50, 80, 120, 240, 360, and 420 min. Next, the aliquot was filtered with 0.45 μm polypropylene filters, and P concentration in the solution was analyzed by ICP-OES spectrometry to determine P in solution. At the same time, the interaction of STR with AHt and citrate in water was explored. With the aim of simulating P, humic and citrate concentrations in soil solution, 30 mg/L P, 30 mg/L C as AHt, and 1 mM sodium citrate were selected to study their interactions in water.^26^ Hence, four solutions with the following compounds were prepared: STR, STR with AHt, STR with sodium citrate, and STR with AHt and sodium citrate. After stirring for 7 h, Mg and P in solution were determined by ICP-OES.

#### 2.4.2 Molecular Modeling of STR in the Presence of Citrate and Humic Acid

Molecular modeling studies were carried out using MM+ force field-molecular mechanics coupled to PM3 semiempirical quantum and the DFT (B3LYP/6-311+G(d,p)) methods, implemented in Hyperchem 8.0 and Gaussian 16W, respectively. Molecular interaction studies were carried out by calculating the stabilization energy after geometry optimization of the system using MM+/PM3, with atomic charges calculated using PM3. The calculation of the stabilization energy of the molecular system was performed by the subtraction of the energy of the molecular system with the molecules placed at noninteraction distances to each other (the energy of the optimized system at a noninteracting distance is equal to single point calculation- and the energy of the optimized system when the molecules are placed at interacting distances to each other. Thus, stabilization energy = energy at interacting distances–energy at noninteraction distances. In order to calculate the intermolecular interactions, different spatial molecular configurations were considered. Only the final conformations corresponding to maximum values of the stabilization energy are presented in the study. Energy values are expressed in terms of the enthalpy of formation (Δ*H*_f_) calculated with PM3 from the optimized structures. To investigate the electronic features (molecular electrostatic potentials, MEP maps) of (i) STR, (ii) citric acid species, and (iii) Temple–Northeastern–Birmingham (TNB), the DFT (B3LYP/6-311+G(d,p)) method was used.

The Temple–Northeastern–Birmingham (TNB) model is extensively used in theoretical studies on the interaction of humic acids with other molecules in water and soil.^27^ This is because the structure contains the main functional groups founded in humic acids of diverse origins. In fact, the use of TNB humic acid in these studies delivered results in good agreement with experiments.^28^ On the other hand, the simplicity of the TNB structure facilitates theoretical studies.

The structures of struvite and their complexes with TNB (−2) and citric acid were optimized with the MM+ force field-molecular mechanics. On the structure obtained, it was calculated the electronic properties, including Δ*H*_f_, by using PM3 semiempirical method. On the structural parameters obtained from PM3, the molecular electrostatic potential map and the molecular electronic density map were calculated from the structure and charges obtained by using DFT (B3LYP/6-311+G(d,p)).

Quantum chemical calculations were performed in the gas phase (in vacuum). This methodology is a simplification of the problem, but we assume a complementarity and correspondence with the results in real conditions.

### 2.5 Analysis of Data

Multiple pairwise comparisons among treatments were made using Fisher’s least significant difference method, with the overall α-level set at 0.05.

## 3 Results and Discussion

### 3.1 Structural Characterization of the STR and STR-AHt

Figure S1 shows the results of SEM-EDX analysis of the STR and STR-AHt samples used in this study. The SEM image of STR reveals well-formed elongated bar crystals with a thin, flat tablet shape, indicative of orthorhombic symmetry and consistent with STR. The composition of these crystals includes P, O, Mg, and N, in coherence with the STR formula ((NH_4_)MgPO_4_·6H_2_O). The SEM image of STR-AHt shows an amorphous morphology with C inserted in these structures, thus indicating the incorporation of AHt into the STR compound. Figure S2 displays FTIR, XRD, and ^31^P NMR, analysis of the used samples. The FTIR spectrum of STR shows peaks at 975, 1350, and 1450 cm^–1^ (Figure S2A). The absorption band at 975 cm^–1^ can be assigned to ionic phosphate, and the weak band corresponding to 1450 cm^–1^ may be due to N–O asymmetric stretching vibration or υ2 NH_4_^+^ modes.^29^ The band at 1350 nm could correspond to symmetric stretching of the nitro group. Relating to the library’s spectra, STR spectra are similar to that of PR, whereas spectra of SSP and MCP share their shape, with BCP having an intermediate aspect. The different similarities are in line with their water-soluble properties, respectively. Regarding STR-AHt, the increase in the peak around 1350 related to STR could correspond to OH deformation and C–O stretching of phenolic OH and COO–antisymmetric stretching, indicating the presence of HS in the STR-AHt compound. XRD diffractogram (Figure S2B) reflects the crystal phases of STR and dittmarite along with calcium and magnesium phosphates. The intensity of the STR peaks is reduced in STR-AHt, which suggests the presence of chemical modifications in the crystalline structures of STR with the introduction of HS, probably by the formation of new chemical bonds, which could explain the apparition of two new peaks (signaled with two arrows in Figure S2B) in STR-AHt. This change also corroborates the amorphous morphology of STR-AHt observed in SEM images. Finally, the solid-state 31P NMR spectrum of STR (Figure S2C) shows a tall singlet peak at 5.94 ppm on the 31P MAS NMR spectrum, which corresponds to phosphate with a shoulder at 4.19 ppm for the heterogeneous crystals. It can also be detected a peak at −8 ppm that could be due to the formation of pyrophosphates by partial hydrolysis of STR. In the spectrum of STR-AHt, it can be detected an increase in the peak at −8 ppm. This peak can be also attributed to phosphate diesters or adenosine and guanosine phosphates^30,31^ both of which could be related to phosphate complexes formed by the presence of humic acid. This is in line with the observed spectra of FTIR and the XRD diffractogram, where magnesium phosphates are detected. Thus, these results confirm both the presence of STR in the selected sample used in the different assays of this study and the inclusion of humic acid in STR-AHt.

### 3.2 Composition of STR and STR-AHt

Nutrient contents of the assayed STR (around 12% P, 10% Mg, and 4% N (Table S1)) are the expected ones compared to similar samples obtained from wastewater treatments. The primary nutrient, phosphorus, is partially soluble in water (17.3% of total P) and totally soluble in neutral ammonium citrate, which represents the organic acids exuded by plants. Thus, it contains plant mobilizable P. In the same way, there is also around 10% magnesium, mainly in the neutral ammonium citrate fraction. Moreover, 4.37% of N is included in this source of P. Therefore, STR contains low water-soluble P, reducing P soil fixation, and high neutral ammonium citrate soluble P, which would supply available P to plants. In addition, the presence of Mg and N would add value to this type of fertilizer. The inclusion of humic acid triggers a slight decrease in P, Mg, and N concentration due to the dilution effect of adding the organic material, but in general terms, the pattern of solubility of the nutrients is maintained.

### 3.3 Agronomic Efficiency of STR and STR-AHt

#### 3.3.1 STR and STR-AHt Soil Incubations

**Figure 1:**
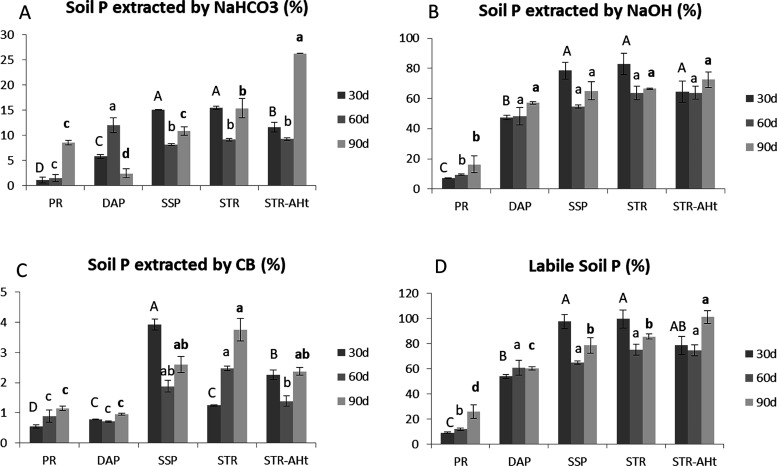
P extracted with NaHCO_3_ (A), NaOH (B), and citrate bicarbonate (C) from PR, DAP, SSP, STR, and STR-AH at 30, 60, and 90 days in bare soil. Sum of the P extracted with the three extractants (Labile-P) (D). Error bars represent the standard deviation calculated from 3 replicates. Bars followed by the same letter are not significantly different at *p* < 0.05. Capital, small, and bold letters correspond to comparisons between treatments at 30, 60, and 90 days, respectively.

**Figure 2:**
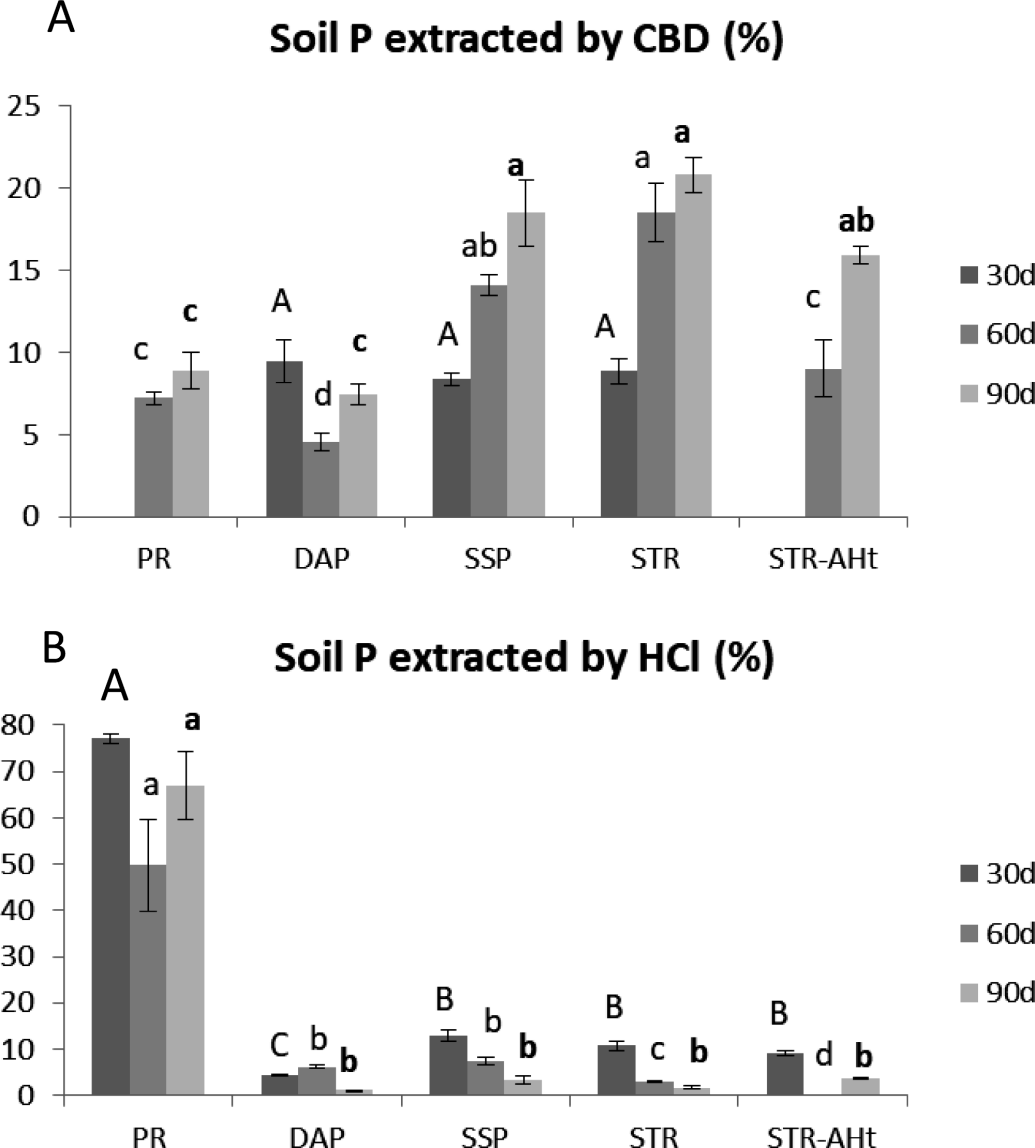
P extracted with citrate-dithionite (CBD) (A) and HCl (B) from PR, DAP, SSP, STR, and STR-AH at 30, 60, and 90 days in bare soil. Error bars represent the standard deviation calculated from 3 replicates. Bars followed by the same letter are not significantly different at *p* < 0.05. Capital, small, and bold letters correspond to comparisons between treatments at 30, 60, and 90 days, respectively.

The interactions of the P-STR with soil compounds are tested in neutral soil with low bioavailable P and low Ca and clay contents (Table 1). Its low P content would favor plant responses to added P. The scarce concentration in Ca and clays suggests a low capacity of that soil to fix P. In that sense, water-soluble sources of P would elude their common disadvantage caused by their high fixing in clays and Ca compounds. In those conditions, high agronomic efficiency of water-soluble P fertilizers is expected, constituting an exigent positive control to evaluate the fertilizer efficiency of STR. Different soil P pools with progressively decreasing bioavailability are obtained, as proposed by Harrell and Wang (2007).^24^ This method involves sequential extraction with five extractants. The BIC-P includes labile P exchangeable by ionic ligands, the NaOH-P extracts P associated with organic matter as well as adsorbed and fixed by Al/Fe oxides,^32^ and CB-P represents the P-pool mobilizable by plant exudates in cases of nutrient deficiency. The sum of the three fractions is considered as labile and potentially bioavailable-P. The CBD-P represents the P occluded in iron oxides, while HCl-P is supposed to be the insoluble Ca–P precipitated as apatite. The sum of these two final steps corresponds to more fixed P in soil. The results obtained in the soil incubation assay are coherent with those of the soil analysis. The low bioavailable Ca and soil clays involve low retention of the different sources of P added. In that sense, total obtained P with the sequential extraction is nearly 100% of the added P for PR, SSP, STR, and STR-AHt (Figures 1 and 2). Along the same line, the concentration of HCl-extracted P, which represents soil-fixed P, is relatively low (Figure 2). Nevertheless, the labile-P values of PR, SSP, STR, and STR-AHt, extracted by bicarbonate, NaOH, and CB, are around 60 and 100% of the added P (Figure 2). The evolution over time of the recovered P of each treatment at 30, 60, and 90 days is compared in each step: P extracted with bicarbonate, the most available for plants and easily exchangeable in soil, is low for PR until 90 days, where almost 10% is obtained; in DAP treatment, there is more P at 30 days, which increases at 60 days and becomes fixed at 90 days; SSP and STR present a similar evolution, with 15% of BIC-P at 30 days followed by a temporary immobilization at 60 days and final restoration of P equilibrium at 90 days (Figure 1A). This is in line with the described trend of phytoavailable P of SSP with time reported by Oliveira et al. (2019).^33^ Generally, the BIC-P values of SSP and STR are higher at 30 and 90 days than those of the rest of the treatments, suggesting a higher available P for plant uptake. When the humic fraction is added to STR, similar or lower values of BIC-P are obtained at 30 and 60 days, but higher values are observed at 90 days passing from 10 to 15% of P from SSP and STR to 25% of P with STR-AHt. Moving to NaOH-P, it represents the major fraction for all of the treatments except PR. As in the prior step, SSP and STR present the major P content with a slight drop with time in both of them. In this case, STR-AHt presents similar values (Figure 1B). The CB-P pool is higher again for SSP and STR related to the rest of the treatments. However, in this case, the evolution is the opposite for them; while CB-P decreases with time for SSP, it increases for STR. The STR-AHt treatment results in terms similar to those of STR and SSP (Figure 1C). This P is associated with the capacity of the crops to mobilize and uptake P in nutrient-deficient situations. Considering these three fractions, it can be observed that labile-P presents a similar profile to the majority P fraction (NaOH-P), with SSP, STR, and STR-AHt generating higher levels of bioavailable P, with a decrease over time in SSP and STR, less marked for STR, whereas STR-AHt provides a bit more concentration of labile-P with time. It accounts for 80, 60, 85, and nearly 100% of the added P for SSP, DAP, STR, and STR-AHt respectively (Figure 1D). Regarding the more fixed P, CBD-P, occluded in iron oxides, is similar for all treatments at 30 days, except for STR-AHt where no P is observed, with an apparent increase with time for SSP STR and STR-AHt (Figure 2A). This trend could be associated with the decreased pattern of SSP and STR in the NaOH-P pool, suggesting a progressive immobilization of P. Finally, HCl-P is the major fraction for PR whereas it remains low for the rest of the treatments where nearly all the P is already extracted in the previous steps (Figure 2B). To sum up, STR and STR-AHt present more bioavailable P than DAP and PR, mainly dissolved in acid conditions, and a similar pattern to SSP, with the only difference in the evolution over time of CB-P and BIC-P, where more P is available with STR and STR-AHt, respectively. The major advantage of STR-AHt over STR is this higher content for BIC-P at 90 days, representing a higher bioavailable P over time. Taken together, these results confirm the hypothesis that in soil with a low fixing capacity of P, STR does not present a significant advantage related to SSP under conditions of soil incubation. Only, when applying STR with AHt, an increase over time in BIC-P can be detected. This is in line with previous works that described beneficial effects on P bioavailability in soils when applying humic complexes of P due to the protector effect of these complexes from soil fixing.^18,34^ The results of this study confirm previous works^35^ that reported the viability of STR as an alternative P-fertilizer in soil incubation studies. In soils with slightly acidic pH, STR shows a behavior similar to that of other P-fertilizer sources, suggesting that it can provide agronomic benefits for crops similar to water-soluble conventional fertilizers. These results also align with those described by Oliveira et al.,^33^ who studied soil P dynamics of a secondary STR in soil compared to SSP through a sequential extraction. They focused on the short-term effect, finding a similar distribution of P forms with SSP and STR, with NaOH-P as the major soil P pool at 30 days of soil incubation. Hence, they concluded that STR improves P-phytoavailability content similarly to commercial fertilizers. They used acidic soil with low Ca and clay content. Nevertheless, they claimed more experiments with diverse soil types and different crops. Thus, this work tries to fill this gap and throw new light on soil P dynamics of STR in different soils and its relationship with crop uptake and yields. In addition, we focus on the long-term effects of STR.

#### 3.3.2 In Vivo Evaluation of STR and STR-AHt: Pot Essays with Barley and Tomato

**Figure 3:**
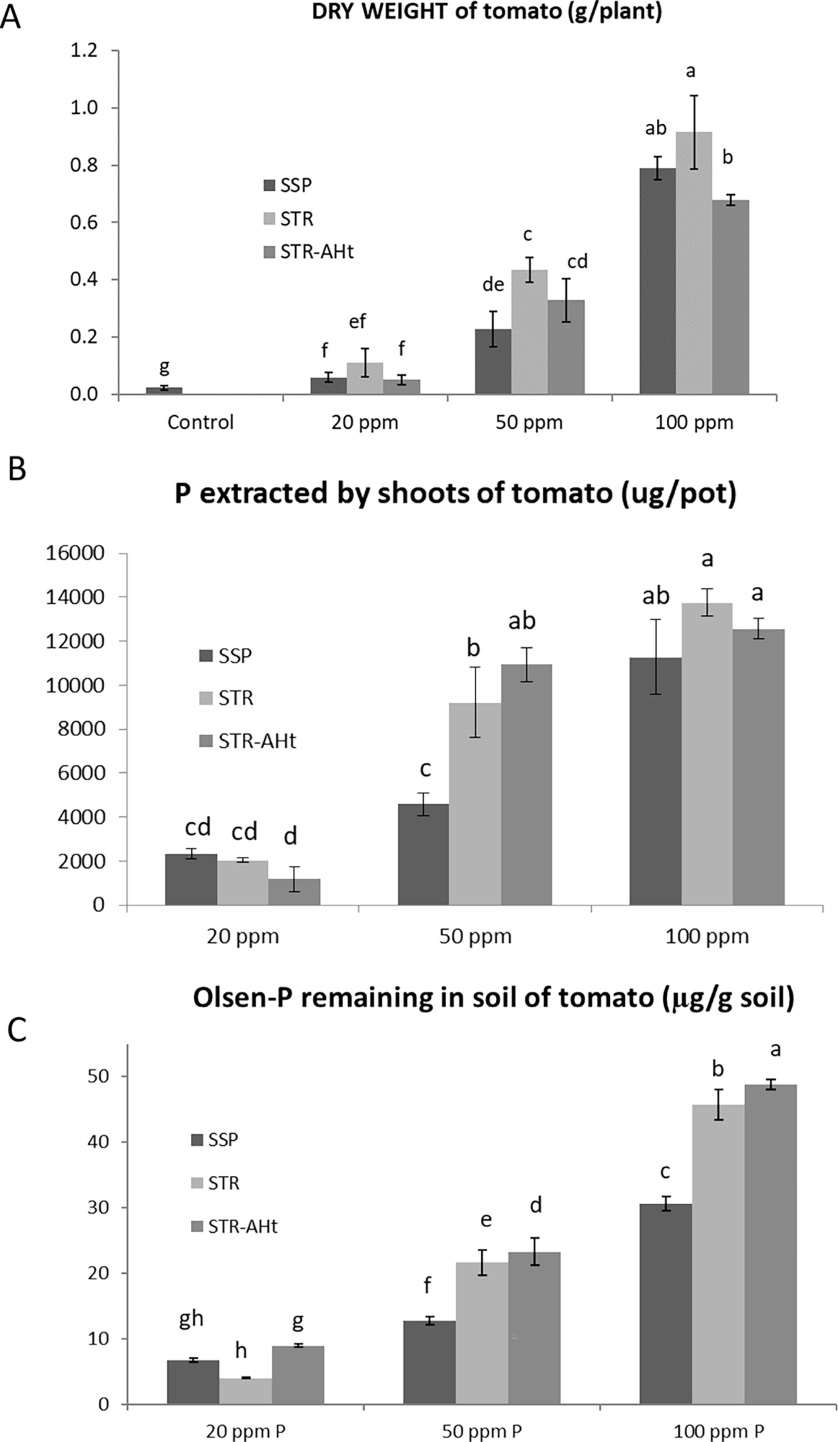
Dry weight (A) and P content (B) in aerial part of tomato treated with SSP, STR, and STR-AHt at different P-doses (ppm). Remaining Olsen-P in soil after cropping (C). Error bars represent the standard deviation calculated from 5 replicates. Bars followed by the same letter are not significantly different at *p* < 0.0.

**Figure 4:**
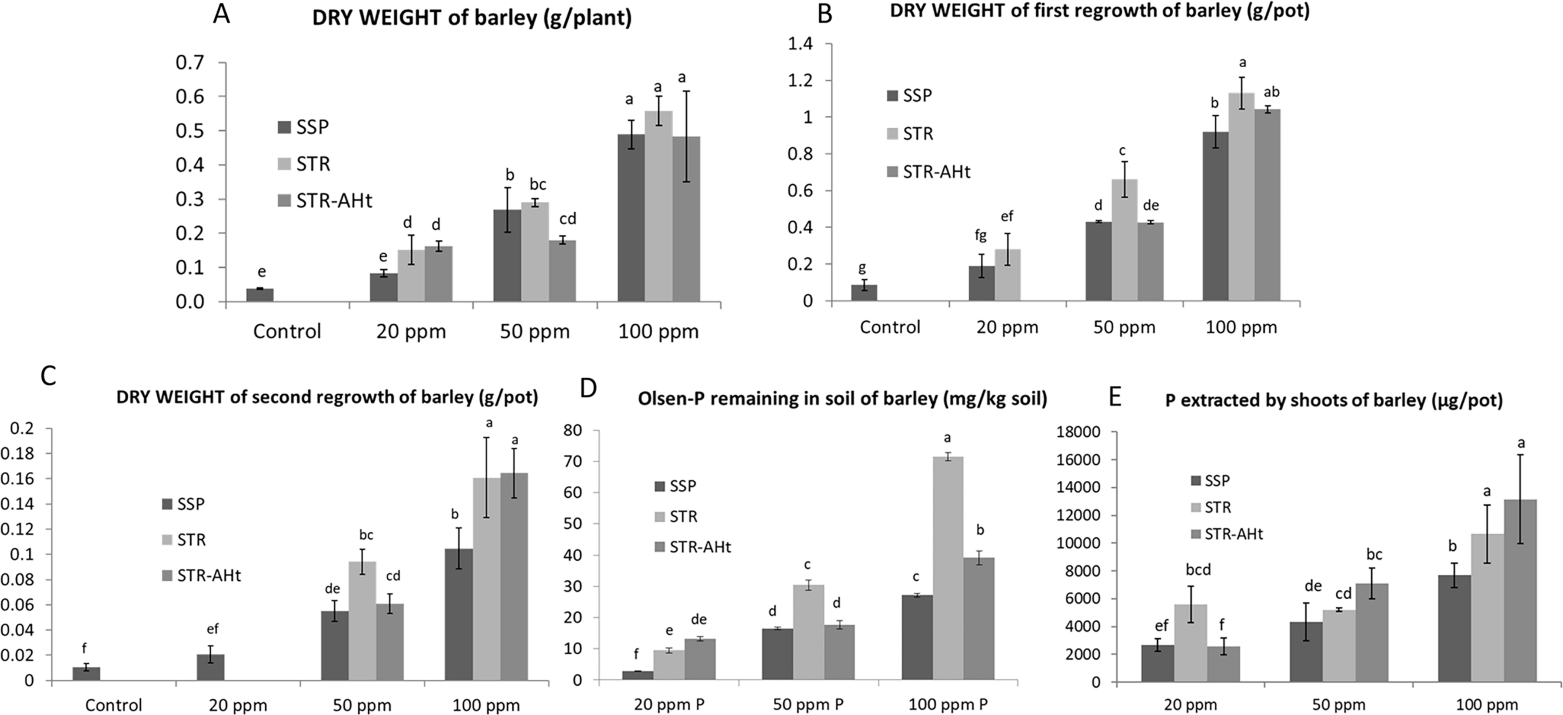
Dry weight at harvest (A) and in first (B) and second (C) regrowth, remaining Olsen-P in soil after cropping (D) and P content in aerial part of barley (e) treated with SSP, STR and STR-AHt at different P-doses (ppm). Error bars represent the standard deviation calculated from 5 replicates. Bars followed by the same letter are not significantly different at *p* < 0.05.

Diverse studies have focused on exploring P fractions in soil with recycled P sources.^36−38^ However, these reports need to validate their results with crop experiments. Likewise, several works have tested the agronomic efficiency of STR without deepening in the evolution of soil P.^39−41^ Thus, pot assays with two different crops are conducted to test the agronomic value of STR and correlate with soil incubation results. In that way, crop yield and P content in the aerial part are determined for tomato and barley when treated with STR, STR-AHt, and SSP. These crops are selected because of their different abilities to mobilize soil P. In that sense, a mono and dicotyledonous crop – tomato and barley – are selected following the assay of Tiziani et al.^42^ Regarding the treatments, SSP is used as a positive reference to evaluate the agronomic potential of STR and STR-AHt according to the results obtained in the soil incubation study, where, whereas similar patterns of P pools with SSP and STR are observed, a slight increase in P bioavailability is detected when using STR with humic acid. In addition, residual P in soil is measured after harvesting. Figure 3A shows the dry weight of the aerial part of the tomato after harvest with different doses of applied P. Related to the untreated plants (control), where no P was added, a clear plant response to the different sources of P at any dose. This result indicates that the selected soil is a suitable one to test the efficiency of P fertilizers. Regarding the different doses of added P, there is a progressive increase in plant yield when the P concentration is increased with both treatments (Figure 3A). Therefore, the comparison in efficiency is carried out in a slope of dose–response of tomato, which shows the suitability of the selected P concentrations. In general terms, STR treatment produces higher yields in aboveground dry matter than SSP at any dose, confirming its potential to supply plant P. At the medium dose of 50 ppm P, tomato aboveground biomass almost doubles with STR, showing significant differences related to SSP (Figure 3A). However, the addition of STR together with humic acid not only does not maintain this improvement but also involves a clear decrease in crop yields compared to STR, specially at 100 ppm. Indeed, compared to SSP, STR-AHt does not suppose higher yields in the aerial part of tomato. These differences between treatments depending on the dose highlight the importance of considering different doses of P to evaluate STR efficiency properly. The P content in the aerial part due to P applications is obtained by subtracting the P content absorbed from the soil in the control plants. Figure 3B shows that the P content in plants follows a trend similar to dry aboveground biomass. The treatment with STR supposes higher P content in plants related to commercial water-soluble P, especially at 50 ppm, with the subsequent improvement in biomass. No beneficial effect of humic acid over STR is observed in terms of P content, being coherent with the aerial part yield observed. Nevertheless, at 50 ppm, STR-AHt results in higher P content in the aerial part than plants fed with SSP, which does not involve a yield increase. Finally, in order to explore the residual effect of STR, the labile-P in the soil after the three harvests was determined using an Olsen extractant. The results show a remarkable store of bioavailable P with STR and STR-AHt at 50 and 100 ppm P (Figure 3C). Around 40% of the applied P remains in soil with the ability to supply P for further potential crops in this soil. In the case of SSP, residual P ranges from 20 to 30% of the applied P. This lower stock of P with SSP together with the lower P content in the aerial part of tomato shows the higher fixed P in soil with SSP. In this line, previous works detected the necessity of performing soil P analysis after cropping to consider the long-term fertilizing capacity of STR.^38,40,43,44^ Taken together, STR treatment triggers a higher P content in the aerial part related to SSP with the subsequent increase in crop yield. In addition, STR application supposes higher bioavailable P stocks in soil for future crops. This fact involves lower P retention in soil, thus reducing the fertilizer impact on soil fertility. The addition of humic acid to STR does not improve plant yields of STR being similar to those obtained with SSP, although higher P contents in plants at 50 ppm are detected. The efficiency of tomato in the mobilization and absorption of insoluble P is in line with the review of Dixon et al. (2020)^45^ that stated the high potential of tomatoes to enhance P use and acquisition by changing P bioavailabilities, allowing the reduction of fertilizer application. In the same way, Tiziani et al. (2023)^42^ reported that tomato exuded organic acids to cope with this deficiency under P depletion conditions. Regarding the assay with barley, as in the case of tomato, it can be appreciated in the aerial part dry weight, a response of the crop to the different applications of P compared to the control without a P supply (Figure 4A). When raising the dose with both sources of P, a consequent increase in plant yield can be observed as being more remarkable when passing from 50 to 100 ppm P. Regarding the fertilizer efficiency of STR, there is a trend in every dose to obtain more yield with STR than with SSP (Figure 4A). In that sense, the lower dose of applied P yielded significantly more biomass production with STR. In line with the trial with tomato, STR-AHt treatment does not improve STR results, with similar plant yields. In contrast with the assay with tomato, the increases due to STR application are less clear in this first harvest. To deepen the residual effect of STR, the first harvest is carried out, cutting the plants 1 cm aboveground, and the experiment is continued watering each week to perform successive harvests. Thus, two regrowth cycles are studied to test the ability of the plants to take up the remaining P in the soil after harvesting. The Olsen-P remaining in the soil after cropping determines this residual effect in the tomato assay. In the case of barley, dry matter production in successive harvests is considered to confirm the bioavailability of soil-P after harvesting. The aboveground biomass of plants in the first regrowth shows the capacity of P from both sources to supply P to plants to keep growing (Figure 4B). The same trend of the first harvest is noted with STR-fed plants, resulting in higher yields, especially at 50 ppm P where barleys treated with STR increase their aerial part weight by around 50% related to plants treated with SSP (Figure 4B). This difference, previously observed at the lower dose of added P, is appreciated in the second harvest at the medium dose of P. In the same way, significative differences between SSP and STR are also appreciated at 100 ppm. Following the same trend, STR-AHt treatment does not involve any advantage yielding similar or even lower production than STR at 50 ppm, showing a similar efficiency as SSP. In the second regrowth, the growth of the majority of plants is kept but to a lesser extent (Figure 4C). In fact, whereas in the previous regrowth, yields are in the range of 0.2 and 1.2 g per pot, in the second regrowth, they range from 0.02 to 0.15 g per pot. In any way, significant increases are maintained for STR plants compared with SSP plants at medium and high doses of P. STR-AHt plants grow at the same level as STR plants at 100 ppm. These results highlight the importance of the residual effect to properly evaluate the fertilizer potential of STR. In this line, high concern is observed in reports about the suitability of conducting long-term trials to evaluate the P release effect of STR.^46^ Equally, this residual effect reflects the lower soil fixation of P from STR compared to the water-soluble P from SSP. This effect permits STR to fit plant demands for extended periods than SSP and triggers less impact on soil fertility, reducing soil salinity, and possible risks of eutrophication. These differences in the two regrowth cycles with STR are coherent with the higher Olsen-P determined in soil after harvesting in the previous study with tomato. In this line, soil-P is also extracted with Olsen after the regrowth of barleys. STR treatments involve higher stocks in the soil at the three doses of P (Figure 4D). On this occasion, STR-AHt presents lower bioavailable P stocks in soil compared with STR. Eventually, Figure 4E shows that the P content in the plant is in line with dry aboveground biomass. In such a way, STR sources suppose higher P content in plants related to SSP, which increases plant yields. No differences are observed between STR and STR-AHt, which is coherent with the corresponding values of dry weights of aerial part. However, as has been commented, when evaluating residual effect through regrowth and Olsen-P remaining in soil, STR-AHt presents lower efficiency than STR. Taken together, the correlation plant assays with soil incubation studies is different than expected. Whereas SSP and STR treatments give similar results in the sequential extraction in soil in the in vitro study, they involve important differences in the plant assays in greenhouse. In addition, when comparing Olsen-P in the incubation study to Olsen-P remaining in the soil in the greenhouse trial, remarkable differences can be observed. This lack of correlation between soil incubation experiments and plant assays was obtained by Talboys et al.,^47^ where the lower P supply in soil by STR gave equivalent rates of P uptake, yield, and fertilizer recovery in plant trials. In the same way, the higher BIC-P at 90 days observed for STR-AHt in the soil incubation study related to the rest of the treatments is not reflected in the plant experiments. This poor correlation between both studies makes the soil incubation design and the sequential extraction of P pools not reliable to predict plant responses with this type of citric but not water-soluble fertilizers that correspond to recycled sources of P. Thus, the influence of the rhizosphere in P mobilization should be considered. With this aim, two studies are carried out to explore the subjacent mechanisms that drive P-STR release in soil as well as the role of humic acid in this P mobilization.

### 3.4 Mechanisms of Action of STR and STR-AHt

In order to explain the results of the agronomic efficiency of STR-AHt, two in vitro experiments of solubility and a molecular modeling study are carried out. The initial hypothesis of improvement of P solubility from STR in the presence of humic acid is supported by the results obtained in the soil incubation essay but discarded by the results described in the pot trials with barley and tomato. Thus, mechanistic studies are required to explain both the unexpected low correlation between soil incubation and pot assays and the no beneficial effect of combining humic acid with STR. With this aim, P-STR release dependence on pH, organic acid, and humic acid is studied in depth.

#### 3.4.1 Nutrient Release from STR in Different Media

**Figure 5:**
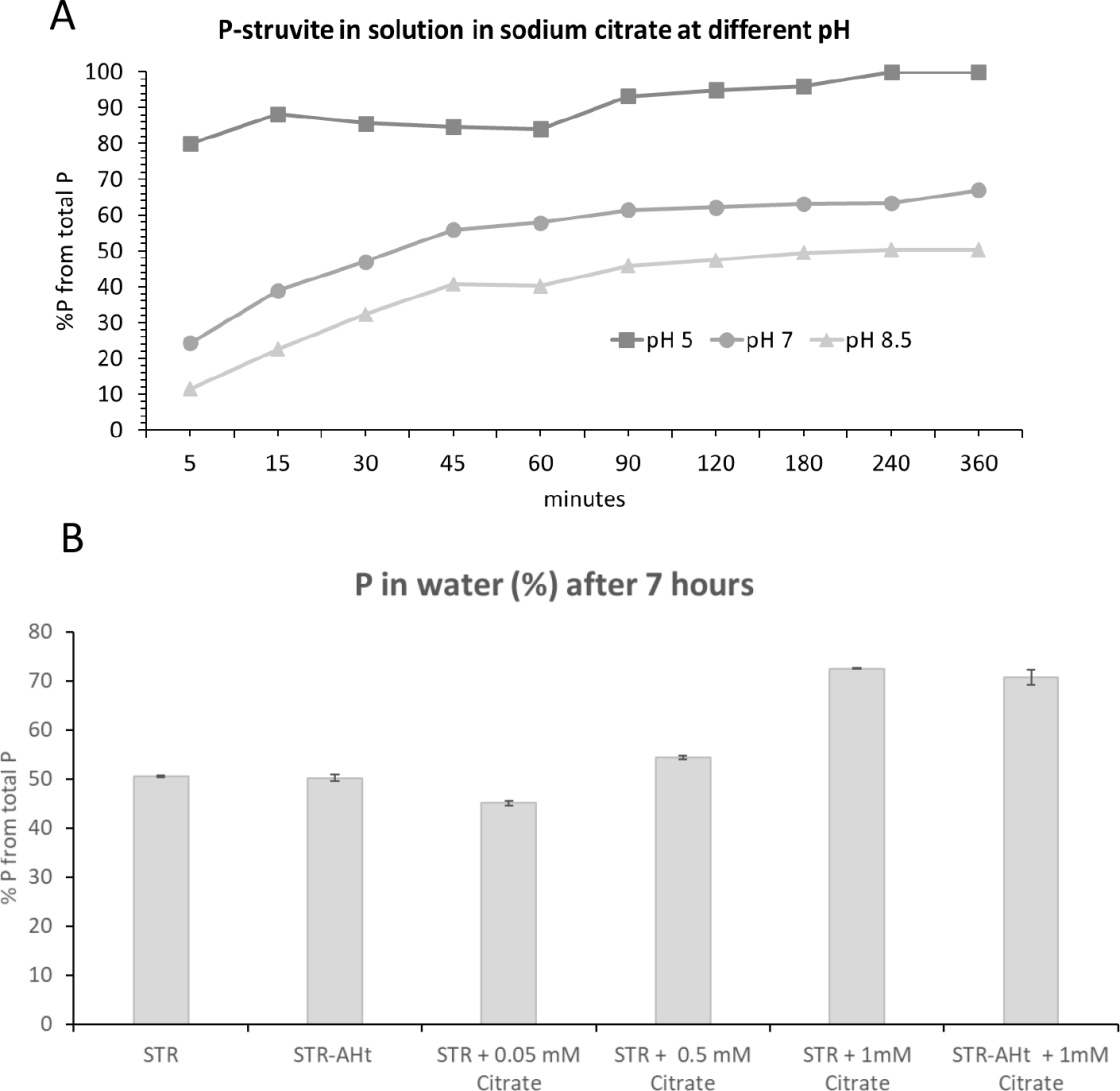
Solubility dynamics of STR in solution: (A) release kinetics of P in sodium citrate at pH 5, 7, and 8.5, and (B) water solubility of P of STR in the presence of AHt, sodium citrate, and AHt with sodium citrate at neutral pH.

Figure 5 shows the solution pattern of P from struvite in citrate and water under different conditions. Figure 5A represents the kinetic curves of P release in sodium citrate at different pH values until the asymptote is reached. It can be observed a gradual increase of P solubilization at pH 7 and 8.5 reaching the maximum value of 60% and 40%, respectively, at 45 min. Regarding P release in citrate at pH 5, 80% of the P in STR is dissolved from the beginning with total release at 4 h. Thus, although there is a partial mobilization of P from STR by the organic acids, the main factor that drives STR solubilization seems to be the pH. Figure 5B describes P-struvite solubility in water after 7 h and the influence of humic acid, citrate, and the conjunction of both. It can be appreciated that whereas humic acid does not increase P water solubility of STR, the addition of citrate at 1 mM triggers an increment of P solubility from 50 to 70% of the total P. However, when applying citrate at the concentrations that can be found in soil solution in the rhizosphere (from 0.05 to 0.5 mM) this increase in P solubility is not significant. This result supports the previous suggestion of considering the pH as the main factor that governs STR solubilization in soil. Thus, it can be concluded that the presence of the plant plays a major role in the configuration of the biochemical properties of soil in its surroundings at, at least, two levels: acidifying the media and raising the microbial activity. Consequently, it should be taken into account to predict bioavailable P in soil. The local acidic atmosphere created by root exudates in their surroundings could increase P solubility from STR, as observed in the release kinetics with citrate at pH 5 related to neutral and basic pH. Therefore, the organic exudates and, especially, the protons secreted by roots would be the major ones responsible for STR solubilization. Regarding the microbial effect produced by plants in the rhizosphere, Velasco-Sanchez et al.^48^ highlighted the influence of soil microorganisms in the solubilization of poorly soluble P forms increasing Olsen-P extraction in soil incubations. In the same way, Ruiz-Navarro et al.^49^ reported the important role of microbes in STR-P solubilization and redistribution into different soil fractions. These microbial communities that influence STR-P dynamics in soils are also affected by STR. Mancho et al.^41^ addressed the positive effect of STR on soil microbes’ activities that support 80–90% of biochemical reactions in soil.

#### 3.4.2 Molecular Modeling of STR in the Presence of Citrate and Humic Acid

##### 3.4.2.1 Chemical Species

In order to investigate the interaction between the STR cell unit and humic acid and root exudates represented by citric acid, the following molecular models are selected. The cell unit of STR consists of the interaction between phosphate (charge −3), Mg coordinated with six water molecules (charge +2), and ammonium (charge +1) (Figure S3). The STR structure is first calculated using molecular mechanics, MM+ force field.^50^ Other force fields are also tested like AMBER^51^ and OPLS^52^ but the results do not respect the crystal structure of the cell unit of STR (data not shown). The results from the calculation are compared with the crystal structural parameters described in Prywer et al.^53^ and are presented in Table S2 and annex 1 of the Supporting Information. Although there are some differences between the calculated parameters and those of the crystal structure, the consistence is reasonably good. The energy-related features of the MM+ optimized structure of STR are calculated using the semiempirical method PM3 and the MEP is derived from DFT (B3LYP/6-311+G(d,p)) atomic charges calculation. To model the citric acid, we use citric acid (charge 0) and citrate (charge −2). This selection is based on some studies that described citric acid chemical species in soil solution.^54^ They established two main forms: citrate (3 Na+) and citrate (Na+). The structure of these species is optimized with molecular mechanics (MM+ force field) and semi-empirical quantum mechanics (ZINDO 1). Due to the problems in optimizing STR-included interactions with ZINDO1, we compare the results obtained for Na+ species with the optimization of citric acid (neutral, charge 0) as equivalent to citrate (3Na+) and citrate (charge −2) as equivalent to citric acid (Na+). The results obtained show that the use of the neutral form of citric acid and the charged form (−2) of citrate is a good proxy to simulate the interaction of citric acid species with the STR cell unit (data not shown). The structures are optimized using molecular mechanics (MM+ force field) and PM3. The MEP of the two species is obtained from DFT-based (B3LYP/6-311+G(d,p)) atomic charges calculation. To model the humic acid molecule the Temple–Northeastern–Birmingham (TNB) structural model is selected.^55^ The structure of TNB (−2) is optimized using MM+ and PM3. The MEP is obtained from DFT-based (B3LYP/6-311+G(d,p)) atomic charge calculation.

##### 3.4.2.2 STR Electronic Characterization and Interactions with TNB Humic Acid and Citric Acid

**Figure 6:**
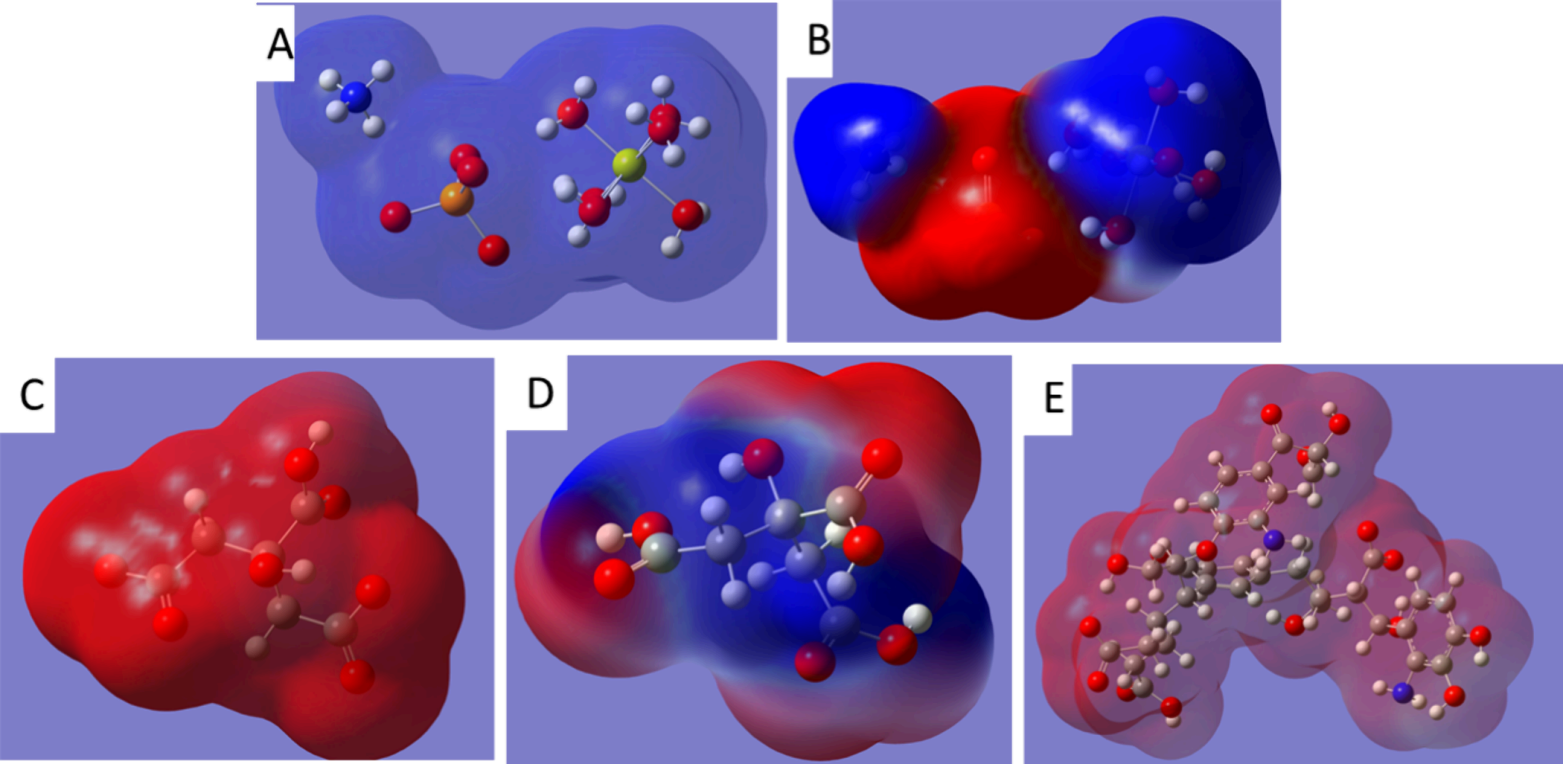
Molecular electronic density (MED) for STR (NH_4_)Mg(PO_4_)·6H_2_O. (A); molecular electrostatic potential (MEP) of STR (B), citric acid(−2) (C), citric acid(0) (D), and TNB humic acid (−2) (E). Negative values in MEP are presented in red and positive values in blue. Element colors: blue: N; white: H; orange: P; red: O; yellow: Mg.

**Figure 7:**
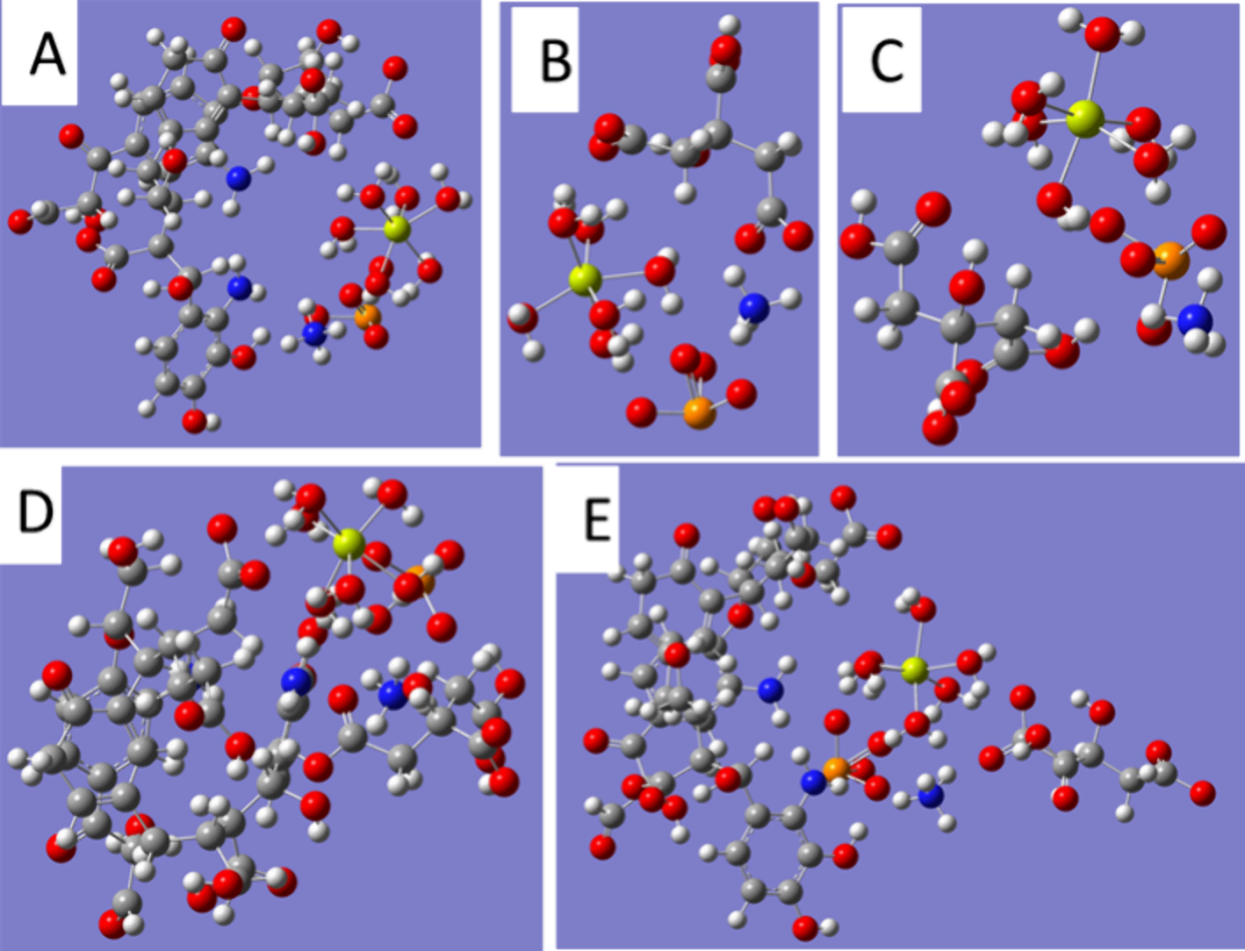
Optimized structures for TNB(−2)–STR (NH_4_)Mg(PO_4_)·6H_2_O (A), citric acid (−2)–STR (B), citric acid (0)–STR (C), TNB(−2)–STR–citric acid(0) (D), and TNB(−2)–STR–citric acid (−2) (E) complexes. Element colors: blue: N; white: H; orange: P; red: O; yellow: Mg.

The energy-related values corresponding to the STR structure calculated with PM3 are presented in Table S2. The value of Δ*H*_f_ is important (−403.258 kcal/mol), presenting a highly stabilized structure. The MED shows a homogeneous distribution throughout the whole STR crystal unit (Figure 6A). The MEP shows a region of negative electrostatic potential around the oxygens of phosphate and a region of positive electrostatic potential around Mg (6H2O) and ammonium (Figure 6B). This configuration shows that negatively charged molecules, such as the humic acid or root-exuded polycarboxylates, might interact with STR through Mg and ammonium. In the case of neutral citric acid (citric acid (0)), the molecule also presents a stabilized structure (Δ*H*_f_= −297.029 kcal/mol) (Table 2) and a MEP distribution showing negative areas around the oxygen atoms of carboxylates and a positive area around the carbon core of the structure (Figure 6C). As it was expected, citrate (−2) presents a stabilized structure (Table 2) with a Δ*H*_f_ = −279.820 kcal/mol and a negative MEP around the molecule (Figure 6D). This negative electrostatic potential favors the nucleophilic attack to STR-positive MEP areas, principally Mg (6H_2_O) (2+) and NH_4_ (+). Finally, TNB (−2) presents high stability (Table 2), with a Δ*H*_f_ value of −673.534 kcal/mol. The MEP of TNB (−2) presents a negative electrostatic potential all around the molecule, showing high potential capacity for nucleophilic attack (Figure 6E). Regarding the interaction of STR with TNB (−2), the results (Table 2) show that TNB (−2) forms a stable complex with STR, without the shift of phosphate from the STR structure (Figure 7A). In this case, the difference between the Δ*H*_f_ value for the interaction distance (−859.663 kcal/mol) and for the no-interaction distance (−831.965 kcal/mol) is −27.698 kcal/mol. The shift of phosphate might occur due to the electrostatic repulsion of negative charges in TNB and phosphate and the interaction of TNB with Mg and ammonium. However, the final result is a stabilization of STR resulting from its interaction with TNB (−2). This fact is consistent with the in vitro experimental results showing that the presence of AH does not increase P solubilization from STR (Figure 5B). The interaction of STR with citric species is also studied. The negative electrostatic potential around all the molecules of citrate (−2) permits the nucleophilic attack to STR positive Mg (6H_2_O) (2+) and NH_4_ (+) areas. As in the case of the STR-TNB(−2) interaction, a potential shift of the phosphate from the STR structure resulting in phosphate mobilization is possible due to the repulsion forces between citrate (−2) negative charges and phosphate negative charges. However, the energy optimization of the molecular system leads to a stabilization of STR structure through the formation of stable complexes between STR and citrate (−2) (Figure 7B). This stabilization is reflected in the value of Δ*H*_f_ for the molecular system for the no-interaction distance (−626.182 kcal/mol) and after the interaction (−663.266 kcal/mol). Thus, the stabilization energy corresponds to −37.084 kcal/mol. The negative areas of carboxylates in citric acid (0) may drive the interaction with the positive areas of STR, mainly around Mg (2+) and NH_4_ (+). Following the same reasoning as that for citrate (−2) and TNB (−2), a phosphate mobilization linked to a shift from STR may occur. However, also in this case, the optimization of the system leads to a stabilization of STR forming a stable complex with citric acid (0) (Figure 7C). In this case, the stabilization energy calculated from the differences of Δ*H*_f_ values for the molecular system in the no-interaction distance (−640.507 kcal/mol) and for the interaction system (−651.246 kcal/mol) is lower than that for citrate (−2) (−10.739 kcal/mol) (Table S2). These results describe a stabilization of STR trough the formation of stable complexes with citric acid species. This is consistent with the experimental results obtained in this study, showing that the interaction of concentrations of citric acid mimicking those present in the rhizosphere does not lead to an increase in phosphate solubilization (Figure 5B). Finally, the interaction of the complex formed by STR and TNB (−2) with the citric acid species is studied. The interaction of TNB(−2)–STR complex with citric acid (0) leads to the formation a stable complex TNB(−2)–STR–citric acid (0) (Figure 7D) with a stabilization energy of −59.883 kcal/mol (Table 2). In the case of citrate (−2), a stable TNB(−2)–STR–citric acid (−2) is also complexed (Figure 7E) with a stabilization energy calculated from the difference between Δ*H*_f_ values for the no-interaction distance (−1123.537 kcal/mol) and the interaction distance (−1143.842 kcal/mol) is −20.305 kcal/mol (Table 2). In summary, these results indicate that the interaction of AH and root exudates with STR at neutral pH would lead to the formation of stable complexes between these molecules and STR and not to the mobilization of phosphate. This conclusion is consistent with the results obtained in this study about the in vitro interaction of AH and citric acid with STR and the consequences of phosphate solubilization (Figure 5B). Likewise, the results are consistent with other studies describing that the precipitation of STR in the presence of dissolved organic matter (DOM) and humic acids led to the formation of STR crystals associated with the organic moiety.^13,15,16^ Finally, the stabilization of STR in the presence of AH or polycarboxylates may lead to a decrease in phosphate mobilization from STR, principally at neutral and alkaline pH.

**Table 2:** Δ*H*_f_ Values for the Different Chemical Species Calculated Using PM3 on the Structure Corresponding to the Energy Optimization of the Molecular System Using MM+/PM3 sp/MM+^a^

**chemical species**	**Δ***H*_f_ (kcal/mol)**(PM3)**
STR	–403.258
TNB (−2)	–673.534
citric acid (−2)	–279.82
citric acid (0)	–297.029
	
TNB(−2)–STR	
NID	–831.965
ID	–859.663
Δ	–27.698
citric acid (−2)–STR	
NID	–626.182
ID	–663.266
Δ	–37.084
citric acid (0)–STR	
NID	–640.507
ID	–651.246
Δ	–10.739
TNB (−2)–STR–citric acid (−2)	
NID	–1123.537
ID	–1143.842
Δ	–20.305
TNB (−2)–STR–citric acid (0)	
NID	–1156.784
ID	–1216.667
Δ	–59.883

a NID means the calculation for no-interaction intermolecular distances, whereas ID is the calculation for the interaction for the intermolecular interaction distances (see material and methods). STR: struvite. TNB: Temple-Northeastern-Birmingham.

In conclusion, the use of humic acids to mobilize the STR is not a feasible strategy to increase the fertilizer potential of STR as it contributes to stabilizing the STR according to the molecular modeling study, which protects P from soil fixation, as can be observed in the higher P labile fraction in the soil incubation, but hinders P release, thus reducing P bioavailability. The key factors that seem to govern STR-like nutrient release are the changes in the pH and microbial environment in the rhizosphere produced by plant exudates. This could explain the low coherence between the results from soil incubation and plant experiments. Therefore, plant action should be considered when using soil incubation to predict the efficiency of STR-like fertilizers by conducting plant assays. In these trials, the residual effect of this type of fertilizer should be studied with long-term assays or by mimicking with the analysis of the remaining P in soil after harvest or by conducting several harvestings to study plant regrowth dynamics. Although the strategy of using humic acids to improve STR fertilizer efficiency has been demonstrated to not be suitable, different alternatives can be obtained from this work to address this issue. Thus, the use of different soil acidifying agents could be a feasible way to increase P release from struvite to fit plant demands in agricultural practices.

## Supporting Information Available

Nutrient contents and solubility of the STR and STR-AHt; bond distances of STR and PM3 calculated structure; SEM-EDX of the STR and STR-AHt; FTIR, XRD and 31PNMR of the STR and STR-AHt; STR structure by molecular modeling (PDF)

## Abbreviations and Nomenclature

STR: struvite
HS: humic substances
AHt: humic acid extracted from peat
SSP: single superphosphate
DAP: diammonium phosphate
PR: rock phosphate
STR-AHt: struvite with humic acid from peat
DTPA: dietilentriaminpentaacetic acid
BIC: sodium bicarbonate
CB: citrate bicarbonate
CBD: citrate bicarbonate dithionite
