# Supplementary material for: Interactions between Struvite and Humic Acid and Consequences on Fertilizer Efficiency in a Nonacidic Soil
Source: J Agric Food Chem. 2024 Sep 18;72(39):21475–87. doi: 10.1021/acs.jafc.4c05472 (PMC11457438; doi:10.1021/acs.jafc.4c05472)
Supplement: Supplementary file 1 — jf4c05472_si_001.pdf [file jf4c05472_si_001.pdf]

Interactions between struvite and humic acid and consequences on fertilizer efficiency in a non-acidic soil

Javier Erro* [a], Iñigo Seminario [b] and José M. García-Mina [a]

[a]Environmental Biology Department. Faculty of Sciences. BIOMA Institute. University of Navarra, c/Irunlarrea, 1, 31008, Pamplona (Spain).

[b]Magnesitas Navarra, S.A, Av. Roncesvalles, 31630, Zubiri, Navarra, Spain

*Email: jerrogar@unav.es

Table S1. Total, bioavailable and soluble content of P and Mg, and total content of N in STR and STR-AHt

	STR			STR-AHt		
**%P soluble in**	**%P_2O_5**	**%Mg**	**%N total**	**%P_2O_5**	**%Mg**	**%N total**
**Total-P (HCl 37%)**	28.3	10.34	4.37	24.0	8.4	
**Bioavailable-P (Neutral Ammonium Citrate)**	27.6	10.32		23.9	8.4	3.51
**Soluble-P (water)**	4.9	0.94		2.9	0.5	
**P- Neutral Ammonium Citrate /Total-P (%)**	97.5	99.8		99.7	99.8	
**P-water/Total-P (%)**	17.3	9.1		12.0	6.2	

Table S2. Bond distances (d) in ångström (Å), comparing struvite crystal structure (crystal) and PM3 calculated on MM+ optimized struvite structure (PM3).

**Chemical bond**	**Crystal (d., Å)**	**PM3 (d., Å)**
P(26)-O(25)	1.55	1.73
P(26)-O(28)	1.54	1.72
P(26)-O(29)	1.54	1.76
Mg(2)-O(5)	2.1	2.03
Mg(2)-O(4)	2.1	2.02
Mg(2)-O(3)	2.07	2.02
Mg(2)-O(7)	2.05	2.02
**Hydrogen bonds O-H…A**	**O-H**	**H…A**
O(5)-H(11) … O(28)		
Crystal (d., Å)	0.81	1.87
PM3 (d, Å)	0.94	1.81
O(6)-H(17)…O(25)		
Crystal (d., Å)	0.78	2.23
PM3 (d., Å)	0.96	2.8
N(22)-H(20)…O(27)		
Crystal (d., Å)	0.9	1.9
PM3 (d., Å)	1.03	1.9

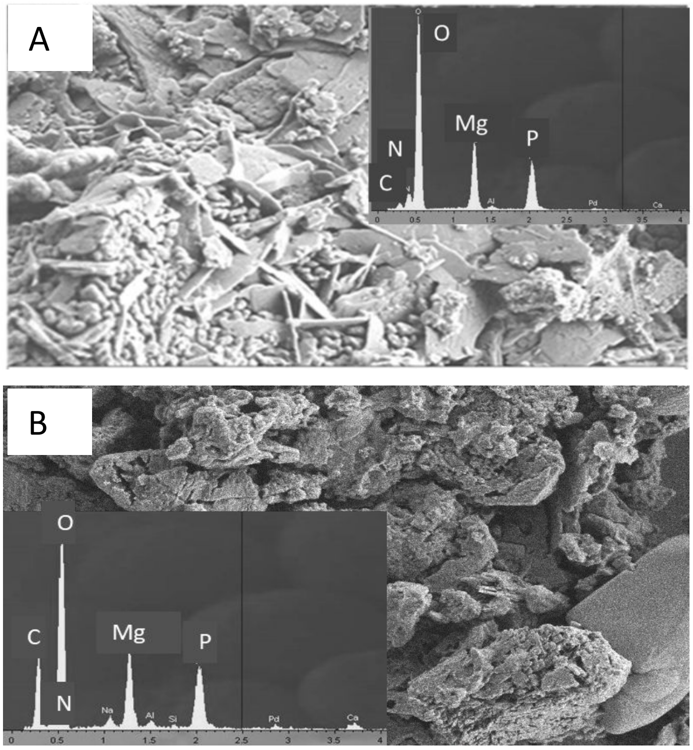

Figure S1. SEM-EDX of (A) STR and (B) STR-AHt.

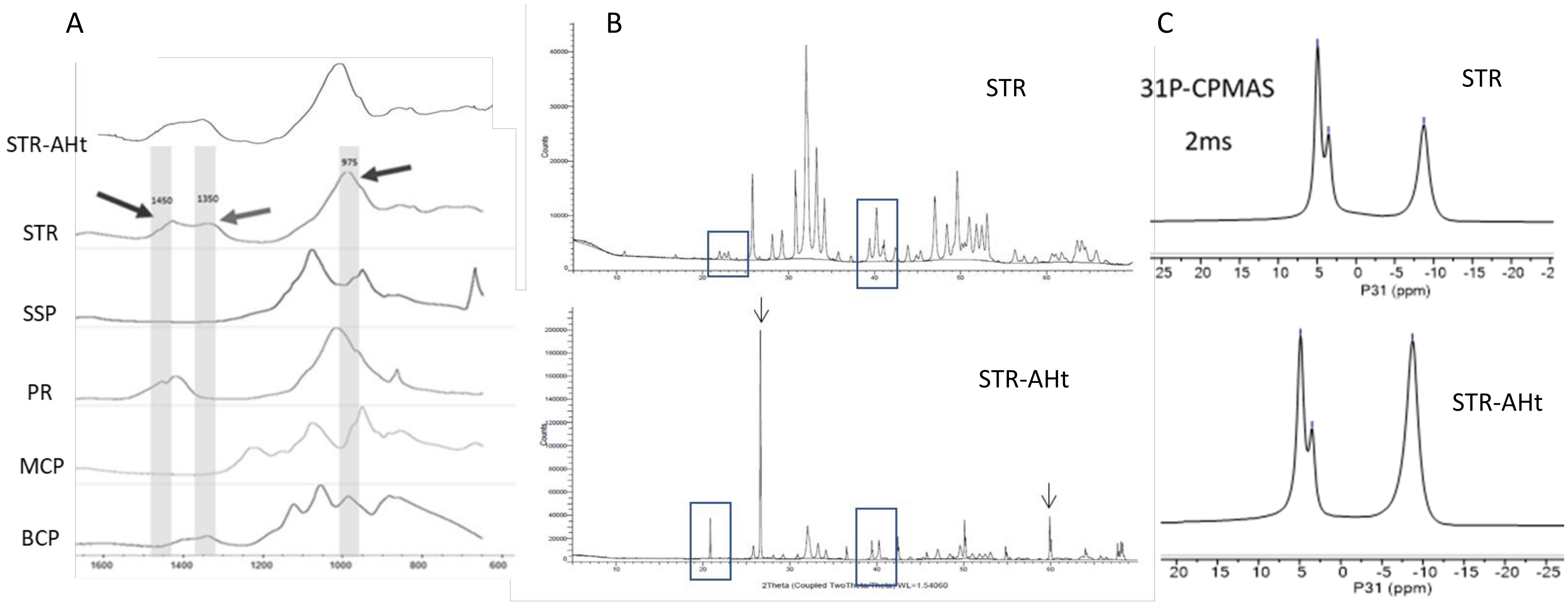

Figure S2. FTIR (A), XRD (B), and 31P NMR (C) of insoluble fraction of STR and STR-AHt

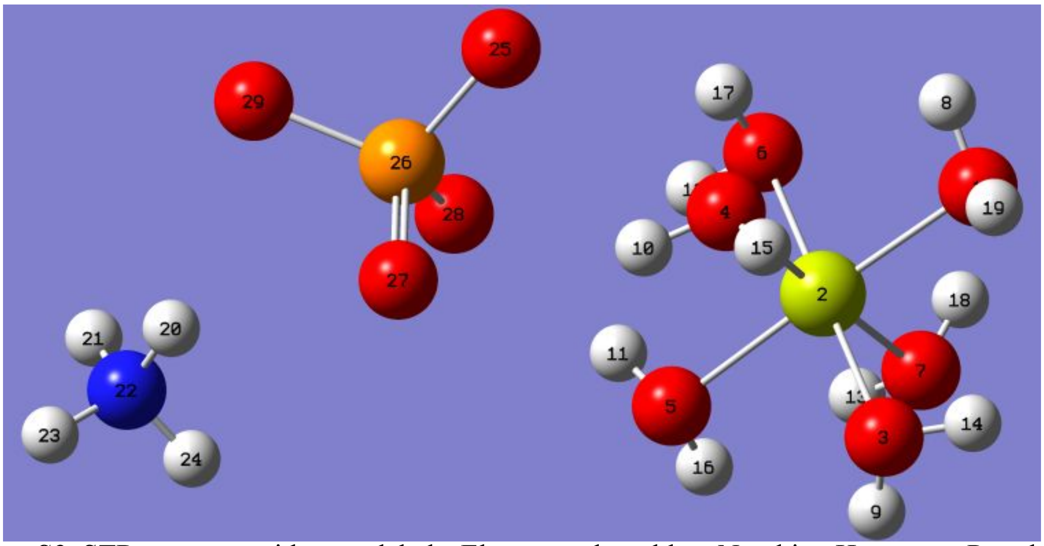

Figure S3. STR structure with atom labels. Element colors: blue: N; white: H; orange: P; red: O; yellow: Mg.
